# Meaning mindset theory: a transdiagnostic approach to mental health promotion and intervention for children

**DOI:** 10.3389/fpsyg.2024.1467332

**Published:** 2025-01-24

**Authors:** Laura Lynne Armstrong, Emmalyne Watt, Catherine Laura Potter, Brent L. Epperson

**Affiliations:** ^1^Saint Paul University, Schools of Counselling, Psychotherapy and Spirituality and Conflict Studies, Ottawa, ON, Canada; ^2^School of Conflict Studies, Saint Paul University, Ottawa, ON, Canada

**Keywords:** meaning mindset, transdiagnostic, mental health promotion, child mental health, third wave positive psychology

## Abstract

A transdiagnostic approach is increasingly recognized as crucial in the prevention or treatment of child internalizing and externalizing mental health concerns. There is substantial overlap and comorbidity among various mental health concerns and the onset of one mental illness elevates the risk of others, underscoring the potential limitations of singular-focused mental health education or treatment. Meaning Mindset Theory (MMT) is a transdiagnostic framework developed and evaluated over the past decade in Canada as a promising new approach. MMT emphasizes agency over thoughts and behaviors, empathy and social competence skills, and meaningful engagement to enhance resilience for both internalizing and externalizing symptoms. The DREAM Program—Developing Resilience through Emotions, Attitudes, and Meaning is a mental health education program grounded in MMT principles. This program has enhanced meaning mindset—agency over thoughts and behaviors, hope for a future that is good, positive self-concept, and openness to learning, new experiences, and feelings—as well as both internalizing and externalizing mental health. To date, the DREAM program, as well as MMT more broadly, has been tested in diverse populations with school-aged children, families, neurodiverse and intellectually gifted young people, homeless men, and Black families, among others. Future research should explore the efficacy of an MMT in therapeutic settings compared to standard treatments, potentially enhancing mental health intervention strategies for Canadian children and families.

## Introduction

In the prevention or early intervention of mental illness in children, a transdiagnostic approach may be most appropriate, broadly targeting both internalizing and externalizing concerns. Specifically, research conducted across the globe suggests that the onset of one mental illness significantly increases the risk of developing another mental illness (McGrath et al., 2020). Further, there are also high rates of comorbidity, a substantial phenotypical overlap between mental illnesses, and subthreshold presentations that can result in social, academic, or home life functional challenges (Dalgleish et al., 2020; Sakiris and Berle, 2019). Therefore, treating a singular concern, or mental health promotion strategies focused on a particular issue, may be inadequate to prevent an overlapping range of mental health concerns or symptoms (Barlow et al., 2017). As mental illness, whether diagnosable or subthreshold, in children and adolescents significantly affects developmental outcomes (Mental Health Commission of Canada, 2024), it is critical to advance strategies that can promote mental health across internalizing and externalizing symptoms.

There are key factors that underlie many mental health concerns. One of these factors is neuroticism, or the propensity to regularly experience intense, challenging emotions, and a sense of uncontrollability or inadequate coping in response to these emotions (Barlow et al., 2021). In fact, the ability to regulate emotions and practice self-control is a critical predictor of life success and future well-being (Côté et al., 2010; Duckworth and Gross, 2014). When strong, challenging emotions are regularly felt, however, people sometimes become skillful at avoiding emotions, given their aversive reactivity to emotional experiences (Sakiris and Berle, 2019). When emotions are avoided, however, this turns off important “signals” that are necessary for distress tolerance.

Imagine emotions as indicator lights on a car: An emotion of joy might signal to a person that this experience is potentially meaningful for them. An experience of fear, for example, may indicate that there is a real threat in the environment and immediate action is needed. Challenging emotions may also signal that someone is having an unhelpful thought. Therefore, emotional avoidance means that someone misses important signals that would have helped them to manage their distress through healthy behaviors or thoughts. Thus, distress intolerance is a core feature underlying many internalizing, externalizing, and personality concerns (Barlow et al., 2021). Emotional avoidance also blunts the experience of positive emotions, enhancing a person’s sense of meaninglessness and unhelpful coping behavior (Moynihan et al., 2022). Overall, experiential avoidance, of which emotional avoidance is a part, is associated with reduced daily positive affective experience (Kashdan et al., 2006). Thus, skills to reduce experiential avoidance, enhancing distress tolerance and agency over thoughts and behaviors to manage feelings, could aid in the promotion of resilience to mental health concerns.

In addition to experiential avoidance—the opposite of openness to new experiences, learning, and feelings—social challenges are also notable across many mental health concerns (Fares-Otero et al., 2021). Specifically, loneliness and feelings of social isolation significantly predict both internalizing and externalizing mental health concerns, as well as poor sleep and risky behaviors (Matthews et al., 2023). Loneliness has been argued to be “trait-like” in nature (Mund and Neyer, 2019), with twin studies demonstrating that loneliness may be partially related to genetic factors, showing a heritability rate of 66% (Goossens et al., 2015; Matthews et al., 2023). Although there seems to be a genetic component to loneliness, at puberty, the social learning environment plays a bigger role in the experience of loneliness and can shape a young person’s experience of social fit in a new direction (Matthews et al., 2023): More specifically, if the factors that underlie loneliness are evident, then teachable skills can enhance confidence and reduce the experience of loneliness. Key factors underlying loneliness appear to relate to empathy (Beadle et al., 2012). An important component to empathy is an ability to attune (Cassidy, 2001). Attunement to others is identified as being a necessary process for healthy relationships, and is theorized to be the facilitator of important relationship skills such as communication and collaboration (Bolis et al., 2022). Attunement involves being aware of, and responsive to, another person’s emotions. The ability to attune can allow for greater adaptive responses and regulation of thoughts and emotions (Bolis et al., 2022). The relationship between emotional regulation and mental health has been extensively researched, with numerous studies documenting that greater emotional regulation is associated with reduction of mental health issues including, but not limited to, depression, anxiety and substance abuse disorders (Berking and Wupperman, 2012). Children and adolescents who have greater emotional regulation skills, are less likely to have later in life externalizing and internalizing issues (Jugmeen and Ciccetti, 2010). However, with some potential deficits in empathy, people who are lonely may not be able to perceive other’s emotional reactions and, thus, rely on their own impressions of their social skills (Beadle et al., 2012). Namely, there tends to be a cognitive perception that one’s social skills are poor even if others would rate their social skills as good (Beadle et al., 2012). Similarly, other research has found that low self-esteem is associated with the experience of loneliness (Okruszek et al., 2023). Further, given that loneliness is associated with neuroticism, lonely people tend to perceive their social aptitude with a negative bias (Beadle et al., 2012). In fact, neuroticism, perceived social problem-solving skills and hopelessness are highly intercorrelated (Walker et al., 2017). Thus, enhancing empathy and perspective-taking skills, as well as teaching skills to build a sense of social competence, countering a negative bias, should ultimately enhance hope, reduce loneliness and, therefore, reduce mental health risk. One perspective that aims to address these issues in a transdiagnostic manner is Meaning Mindset Theory (MMT or Meaning Mindset Therapy): a Canadian approach.

## Meaning mindset theory

A sense of meaning—through creativity or helping behavior, valued activity engagement and connection with others (to other humans, to nature, or to a deity), and healthy thoughts toward situations—is an important predictor of emotional, social, and behavioral mental health (Frankl, 1946/1986; Ivtzan et al., 2015; St John et al., 2024). Moreover, meaning is a key factor in promoting resilience to future difficulties (Wong, 2017). The earliest pioneer of a meaning-based theory for mental health, Frankl (1946/1986), posited two different conceptualizations of meaning. Firstly, people may experience a sense of an overarching purpose in life. Secondly, one may perceive moments and experiences that are meaningful in everyday life (Frankl, 1946/1986). It is the daily sense of meaning that is particularly associated with mental health and is also sensitive to change through learning in comparison to overarching purpose in life (Armstrong et al., 2019; Frankl, 1946/1986). Research suggests that skills to foster perceived meaning in daily life can be acquired (Armstrong et al., 2019; Frankl, 1946/1986). Namely, these skills are built through cultivating a Meaning Mindset (MM). MM is an orientation toward noticing *valued* experiences, connections, or situations. It is the recognition of a spark of awe found in nature, art, music, and human connections or a self-transcendent feeling (e.g., mindfulness, flow, or a perceived spiritual experience; Frankl, 1946/1986; Keltner, 2023; Shiota et al., 2014). The meaningful moments found in experiences, connections, and helpful thinking are felt when there is perceived agency over thoughts and behaviors; openness to new experiences, to learning, to one’s own and other’s emotions; positive social self-concept and perceived ability to set and reach achievable goals; hope for a future that is good (Armstrong et al., 2018). This skillset of agency, openness, positive self-concept, and hope is MM.

**Figure 1:**
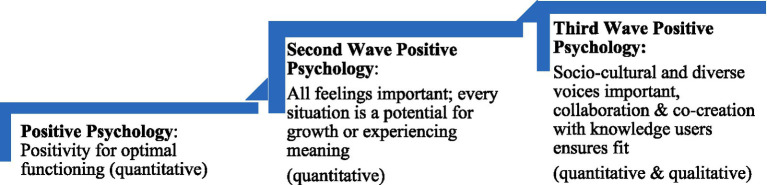
Evolution of positive psychology (bio-psycho-social-ecological well-being) – based on Lomas et al. (2020).

MMT is a Third Wave Positive Psychology (PP3.0) theoretical approach. It is a community action framework for the conceptualization and development of mental health education and treatment, research, questionnaire design, as well as program or protocol development and evaluation (Armstrong and Potter, 2022). Regarding PP3.0, this perspective builds on Second Wave Positive Psychology (PP2.0), which has its foundations in existentialism and Frankl’s Logotherapy (Frankl, 1946/1986; Wong, 2017). In PP2.0, both agreeable and challenging feelings and situations are critical in the expedition toward meaning and mental health (Ivtzan et al., 2015; Wong, 2017). As noted, even difficult emotions can be helpful signals for threats or suggest the presence of an unhelpful thought. Emotional suppression can make change difficult and prevent the achievement of optimal well-being (Ivtzan et al., 2015). Differing from first wave Positive Psychology, in which the seeking of pleasurable emotions and happiness is aimed at well-being, PP2.0 posits that happiness is the generated biproduct of discovering meaning in daily life. Adding to PP2.0, PP3.0 (Lomas et al., 2020) acknowledges the social or environmental systems and groups in which a person dwells: Each individual has their own history of experiences, values, beliefs, and biases that they bring to situations. PP3.0 is, therefore, multidisciplinary and diversity-friendly, uses qualitative and quantitative research, and it aims to have social justice implications (Lomas et al., 2020). This framework engages diverse voices, through which the definition of well-being may look different depending on the social, cultural, or environmental context, fitting well with a transdiagnostic perspective. Further, PP3.0 involves ethical practice guided by values, principles, responsibility, and personal strengths (Lomas et al., 2020; Wissing, 2022). Thus, as a research method, MMT, under the umbrella of PP3.0, uses a Knowledge Translation Integrated (KTI) approach involving co-creation with knowledge-users to ensure “fit,” as well as equity, diversity, inclusivity, and belonging, through the scientific standards of acceptability, credibility, sustainability, and feasibility (Armstrong and Potter, 2022) (see Figure 1).

As noted, MMT is under the umbrella of PP3.0, building on PP2.0 (Armstrong and Potter, 2022; Armstrong and Potter, 2023). MMT was developed and tested over the past decade through ongoing collaboration with knowledge-users, namely over 200 children, parents, school board mental health teams, and mental health practitioners, such as psychologists, psychiatrists, and psychotherapists. This collaboration occurred to ensure the fit of the approach and to establish its credibility, acceptability, feasibility, and sustainability for those who would use or participate in MMT programs. Further, over twenty graduate students have tested and used this theoretical framework in their thesis research with diverse school children, blended families, intellectually gifted children at risk for mental health concerns, homeless men, Black parents, and families on mental health waitlists, among others. MMT has also been developed as a conflict coaching protocol called “EASE” (Empathic listening: reflect, summarise, explore content and feelings with an attitude of curiosity and openness; Acknowledge circumstances: what can be changed and what cannot; Sensible action: help person explore options, problem-solve a plan, foster hope; Explore thoughts and feelings: address difficult feelings and reduce likelihood of inaction) for ombuds practice in order to build alliance, ground the profession in a theoretical, testable framework, and aim to lead to positive conflict resolution outcomes (Armstrong and Epperson, 2024; Epperson and Armstrong, 2024).

Originally called REAL, MMT had its foundations in Rational Emotive, Attachment and social skill-building theories, and Logotherapy (Armstrong, 2016; St John, 2017; Armstrong et al., 2018, etc.). Rational Emotive Behavior Therapy is a cognitive-behavioral transdiagnostic approach to building agency over thoughts and behaviors through addressing should and must thoughts, as well as awfulizing thinking (Dobson, 2010; Ellis, 2004). Attachment-building skills in MMT are centred in socio-emotional literacy approaches (Lynch and Simpson, 2010). Learning experientially through play is central to children’s development of attachment with others (Gil, 1994). Further, attachment through attunement to other’s feelings has been identified as fundamental for healthy relationships (Leerkes et al., 2009), and for optimal attachment (Rees, 2007). Secure attachment with others is linked to developmental outcomes such interpersonal skills and self-regulation (Leerkes et al., 2009; Di Renzo et al., 2020). Furthermore, insecure attachment, which is associated with low attunement, can lead children to potentially adopt more characteristics associated with loneliness and social avoidance (Rees, 2007). In addition to attachment, the meaning mindset framework stems from Frankl’s Logotherapy. Thus, elaborating on the four key tenets introduced previously, MMT’s grounding in positive psychology and its roots in Rational Emotive, Attachment, and Logotherapy perspectives can be seen below:

- Agency over thoughts and behaviors: The belief that one can evaluate their thoughts and can take responsibility for their actions. Healthy thinking and behavior can in turn regulate emotions. A person can choose their attitude under almost any circumstance (Frankl, 1946/1986). Feelings are signals, and an awareness of these signals, pulls for greater engagement in positive actions that spark joy, moving away from harmful actions or situations, processing of difficult experiences, and evaluating unhelpful thoughts.
- Positive self-concept: Building of Dweck’s (2015) growth mindset, positive self-concept within MMT is the belief that one is capable of setting and achieving goals though effort, using effective strategies, and asking for help from others, when needed. A positive self-concept permits one to launch beyond ‘survival mode’ to perceiving everyday meaning in life (Van Tongeren and Green, 2010). Positive self-concept also includes perceived social competence. Specifically, perceived social competence includes the awareness that a person can recognize one’s own and other’s feelings and that different people—including oneself—can feel differently in the same situation. It involves the perceived ability to evaluate social situations and adapt, selecting appropriate social behaviors for a given context (APA Dictionary of Psychology, n.d.).
- Openness to experience: Openness is an attitude of curiosity and a desire to experience new things and to learn. It also includes an aspiration to observe one’s own and other’s feelings, distress tolerance, as well as to be mindfully present in enjoyable activities. Openness allows for meaning to be perceived in connection with others, in learning and in creative pursuits, and in appreciation of experiences through sparks of flow, awe, or gratitude (Frankl, 1946/1986; Keltner, 2023; Shantall, 1989; Stoddard et al., 2011).
- Hope for the future: This concept involves an anticipation of a future that is good, even in spite of difficult current circumstances, and a sense of the possible: A person who has a ‘why’ or a ‘what for’ can often bear almost any ‘now’ (Frankl, 1946/1986; Nietzsche, 1889/2003; Stoddard et al., 2011). Hope is built through a focus on what is within one’s control instead of dwelling on the uncontrollable (Brilakis, 2021). Hope also involves grit: persistence in the face of challenges, optimism about possibilities, and challenges perceived as opportunities to grow and learn, rather than obstacles (Duckworth, 2016). This is the opposite of perfectionism: Instead of trying to be perfect or avoid failure, every completed task or activity can be viewed as an opportunity for growth and learning with aspects that one is proud of and with things that one might do differently the next time. As rocket scientist Sylvia Acevedo has said, F.A.I.L. equals “first attempt in learning” (Stanford eCorner, 2020). Hope for the future also involves engagement in regular, meaningful activities, and having things to look forward to (Armstrong and Manion, 2015).

The teaching of MMT strategies in various educational and therapeutic populations (e.g., families on mental health waitlists, blended families, children, neurodiverse persons, and homeless persons) has been evaluated and it was found to enhance meaning in daily life, as well as both internalizing and externalizing mental health, in comparison to controls (e.g., Armstrong et al., 2018; Champaigne-Klassen, 2024; Desson, 2018; Fabes, 2024; Odenigbo, 2023; Potter, 2022; Watt, 2020). MMT explores mental health from attitudinal, behavioral, social–emotional competency, meaning-centred, and societal-context angles (Armstrong, 2017). Namely, self-regulation through healthy thinking and actions enables a person to get along better with other people, a skill that is key for resilience to mental illness (Baumeister et al., 2009). The social–emotional components in MMT aim to build perspective-taking and social problem-solving skills to develop empathy and build secure attachment with others (Armstrong et al., 2019; Schonert-Reichl et al., 2015). In MMT, secure attachment is a core pathway to experiencing meaning in daily life and mental health (Armstrong, 2017). The meaning-centred elements in MMT conceive that meaning exists under all circumstances, and that one must discover the meaning potential of each situation (Frankl, 1946/1986). Building on Logotherapy’s pathways to meaning, MMT’s paths to meaning include: (1) creativity or helping others, (2) learning skills to developing secure connections with others or experiencing awe, joyous moments in connection with something beyond oneself (e.g., nature, activity engagement), or gratitude, and (3) choosing healthy thoughts and actions, while perceiving oneself as competent and capable to do so. Regarding hope, in MMT, it involves recognizing and choosing things that trigger feelings of joy, focusing on what is controllable and problem-solving, and viewing challenges or obstacles as opportunities for growth.

In MMT, the four components of MM—agency, self-concept, hope, and openness—can be operationalized through the CHANGE model, which is the mechanism of action through which “change” occurs (Armstrong and Potter, 2022; Armstrong and Potter, 2023; Epperson and Armstrong, 2024):

- *Challenge unhelpful thoughts*. In response to challenging emotional signals that suggest unhelpful thinking, teach skills to evaluate thoughts: e.g., What is the best thing that could happen? The worst? What is the likelihood of the best or worst thing happening? What can we do to increase the likelihood of the best thing happening? Externalize: What would I say to a friend if they said this thing to me? Can I ask someone for more information or otherwise seek out more information?
- *Healthy actions*. Scheduling of feel-good or calm-down activities. Teach how to set small, achievable goals, and create an action plan. Explore unhelpful thoughts or predictable triggers for difficulties in reaching goals and use calm down strategies or problem-solving skills to address challenges.
- *Acknowledge circumstance*. Reducing avoidance behaviors of denial or resistance by learning to focus on what can be changed versus what cannot be changed. Using energy for things that can be changed: Is there some small thing that I can do that might be helpful in this situation (moving from disempowerment to empowerment)?
- *Need for belonging and self-compassion*. The most important pathway to meaning involves experiencing connection through relationships with others (Frankl, 1946/1986). Relationships are important for defining self-identity and developing a positive self-concept. Social skills, such as perspective-taking skills, conflict resolution, and emotional literacy, are important for a sense of belonging. Self-compassion skills are also important for a positive self-concept. Practicing positive, realistic self-talk can foster self-compassion: What could you say to a friend who is putting themselves down? Could you say these things to yourself? What are some brief, enjoyable or relaxing activities that you can do to take care of yourself when you are struggling or to use in order to keep feeling okay?
- *Gratitude*. A realistic attitude of gratitude can help a person find daily meaningful moments. Practicing gratitude through gratitude journal or writing letters of gratitude, for example, can also be helpful to build a sense of hope and improve mental health (Allen, 2018).
- *Emotional language*. All feelings are helpful signals, as previously noted. Emotional literacy is, therefore, a key foundation for openness to experience or for recognition that an unhelpful thought may be triggering a challenging feeling. People can be taught to notice and monitor their feelings: What is going on for me right now?

Although MMT has been tested with various populations over the past decade, following the CHANGE model to effect change in MM and mental health, it has primarily been used with diverse groups of school-aged children, including neurotypical, neurodiverse, and intellectually gifted children. Specifically, MMT has been used for mental health education in the classroom, in community clinics, and virtually, following the DREAM Program protocol (see Tables 1, 2).

**Table 1:** MMT in recent research.

Population	Key findings	Statistical results	Citation
DREAM program			
Intellectually gifted children & their parents (6–12)	1) The relationship between gifted oversensitivities and mental illness symptoms was fully mediated by meaning mindset. 2) MM significantly improved from pre-test to post-test in parents and children participating in the DREAM program.	1) The regression yielded an R^2^ of 0.84 at Step 1 (*β* = 0.92, *t* = 4.03, *p* = 0.03, partial eta^2^ = 0.92) for the prediction of mental illness symptoms by gifted oversensitivities. With the addition of MM in Step 2, the relationship between gifted challenges and mental illness symptoms was non-significant, *β* = 0.62, *t* = 0.99, *p* = 0.43, partial eta^2^ = 0.26. 2) *t*_(12)_ = 1.699 *p* = 0.05, A paired sample effect size analysis using Cohen’s d indicated a moderate effect size (*d* = 0.50).	Champaigne-Klassen (2024)
School children during the pandemic (6–12)	1) Meaning mindset significantly increased from pre- to post-administration of the DREAM program. 2) A change in meaning mindset scores inversely predicted mental illness symptom and predicted positive COVID-19 coping.	1) The results of a paired samples t-test (*t* = −8.11, *p* < 0.001, CI_95_ = −16.72, −10.00) between pre- and post-test MM results yielded a large effect size, Cohen’s *d* = −1.41. 2) *F*_(1, 31)_ = 27.91, *p* < 44 0.001, *ηp^2^* = −0.69. and *F*_(1, 31)_ = 15.40, *p* < 0.001, *ηp^2^* = 0.58, respectively.	Potter (2022)
School children (6–12)	MM predicted mental health	The regression indicated (*R^2^* = 0.50) that half of the variability in mental health scores was explained by meaning. The analysis showed that meaning scores significantly predicted internalizing and externalizing mental health scores (*β* = 0.90, *p* = 0.001).	St John et al. (2024)
Families on mental health waitlists	1) There was an increase in parent and child self-reported MM from pre-test to post-test DREAM program administration 2) Meaning mindset inversely predicted mental illness symptoms	1) The analysis of MM yielded significant differences between the pre-test (*M* = 86.50, *SD* = 30.56) and post-test scores (*M* = 101.25, *SD* = 27.43), *t =* −2.628, *p* < 0.023, *df* = 11, *d* = −0.43 2) *F*_(1, 12)_ = 8.42, *p* = 0.01, *ηp2* = −0.64.	Watt (2020)
Intellectually gifted school children (6–12)	1) MM significantly improved from pre-test to post-test DREAM administration 2) A change in MM scores predicted a change in mental health scores	1) *t*_(33)_ = −4.20, *p* < 0.001, *d* = −0.738. 2) The regression yielded an *R^2^_change_* of 0.32, which indicates that a third of the variability in mental health scores was explained by a change in meaning scores (*β* = 0.66, *F_change_* = 21.79, *p* < 0.001). When meaning scores change by one-point, mental health scores change by 0.66. Beta coefficients in step two for the predictors were *β* = 0.135, *t* = 0.995, *p* = 0.347, partial *eta^2^* = 0.12 MM. pre-test and *β* = 0.661, t = 4.668, *p* < 0.001, partial *eta^2^* = 0.56.	Armstrong et al. (2018)
School children (6–12)	MM scores were significantly higher from pre-test to post-test, but were non-significant for the control group (mental health promotion as usual in the classroom)	*t* = −3.30, *p* = 0.006, CI_95_ = −14.17, −2.90, *d* = 0.99. Pre-test and post-test means were 90.69 (sd = 7.23) and 99.23 (sd = 9.92), respectively. Control group, *p* > 0.05.	Armstrong, 2017
MMT applied with other samples			
Homeless men	Pre-post results suggest a single session meaning-based program significantly enhanced MM in homeless men.	*t*_(35)_ = −12.306, *p* < 0.001, *d* = 1.467	Fabes (2024)
Lebanese women	MM mediated the relationship between misalignment in sexual honour beliefs and permissive actions with mental health.	*R^2^_change_* = 0.27 at step one, which indicates that over a quarter of the variability in mental illness symptom scores was explained by alignment scores, (*β* = −0.52, *F*_change_ = 13.65, *p* < 0.001, *t* = −3.70, *p* < 0.001, partial *eta^2^* = −0.52 medium effect size). In step two, *R^2^_change_* = 0.08 (Fchange = 4.00, *p* < 0.05). Beta coefficients in step two for the predictors were: Alignment *β* = −0.35, *t* = −2.19, *p* < 0.05, partial *eta^2^* = −0.29 (small effect size); MM *β* = −0.32, *t* = −2.00, *p* < 0.05, partial *eta^2^* = 0.27 (small effect size). Partial eta2 for alignment was reduced from −0.52 to −0.29 with the introduction of the MM variable in step two.	Halabi (2023)
Adults with ADHD	MM mediated the relationship between ADHD symptoms and intuitive eating	*R* = = −0.11, 95% CI = −0.25, 0.01.	Ciccarelli (2024)
Black mothers	The relationship between parental racial stress and child mental illness symptoms was mediated by child MM	Step 1 (parental racial stress) *R*^2^ = 0.20, *F*(1, 19) = 4.79, *p* = 0.04, χ2 = 0.45, ß = 0.45; Step 2 (child meaning mindset) *R*^2^ = 0.28, *F*(1, 18) = 3.49, *p* = 0.04, χ2 = −0.28, ß = −0.32. In Step 2, the relationship between parental racial stress and child mental illness symptoms was rendered non-significant, *R*^2^ = 0.28, *F*(1, 19) =4.79, *p* = 0.21, χ2 = 0.26, ß = 0.30.	Odenigbo (2023)

**Table 2:** DREAM framework of skills (skills below are taught with webisodes, original songs, and hands-on art, drama, read-aloud stories, and game activities).

Identification & management of emotions	Stress management, coping & perseverance	Healthy relationships	Self-awareness & sense of identity	Critical & creative thinking
Identifying common feelings	Identifying healthy actions	Responding to other’s feelings	Respect for difference	Choice & responsibility
Feeling “signals” to recognize unhelpful thoughts & behaviors	Asking for help	Behaviors that affect self & others	What makes us unique & alike	Making reasoned decisions
Bodily-felt emotions	Healthy & unhealthy use of social media	Non-verbal emotional cues	Cultivating a meaning mindset	Identifying & challenging unhelpful thoughts
Calm-down activities to manage feelings	Managing worries, avoidance, & disappointment	Listening actively	“Me to we” actions	Identifying & solving problems
Relaxation strategies to manage feelings	Importance of, & strategies for, healthy sleep	Perspective-taking	Building gratitude	Fostering grit

### MMT compared to other evidence-based inteventions

MMT has its foundations in Rational Emotive Behavior Theory (REBT), Attachment Theory (AT), and Logotherapy. REBT developed by Albert Ellis, is a Cognitive-Behavioral approach aimed at addressing problematic thoughts, emotions, and behaviors. REBT emphasizes that irrational beliefs, such as rigid “shoulds” and catastrophic thinking, obscure the experience of emotions like pain and joy, often leading to unhealthy anger, anxiety, or depressive symptoms (Ellis, 2004). By fostering adaptive interpretations and problem-solving strategies, REBT empowers individuals to take responsibility for their mental well-being. Techniques such as relaxation and behavioral activation enhance self-regulation, facilitating better relationships and resilience against mental illness (Baumeister et al., 2009). MMT teaches healthy thinking through recognizing emotional signals for unhelpful thoughts and challenging unhelpful thoughts through evaluating them and externalizing them.

Attachment theory highlights the role of empathy in fostering secure relationships and mental health. Strategies like perspective-taking, social problem-solving, and creative play build empathy and connection (Lynch and Simpson, 2010; Malchiodi and Crenshaw, 2014; Schonert-Reichl et al., 2015). Additionally, engagement in community activities and problem-solving fosters a sense of belonging, motivation, and academic success, reinforcing mental health and attachment to others (Billig, 2000; KidsMatter, 2017). In MMT, experiential activities are used to build connections between people, as play is the language of connection (Gil, 1994). Further, through perspective-taking and social problem solving, in MMT externalizing unhelpful thoughts (e.g., what’s a different feeling that someone might have in this same situation? Why might they feel different?) can help people to see that different people may feel, think, and act differently in the same situation. Further, if people can have a different experience in the same situation, it can allow for a person to realise that they themselves could feel, think, and act differently in the same situation.

Logotherapy, created by Frankl, is an existential approach that focuses on meaning-making under all circumstances. It teaches that individuals have the freedom and responsibility to make meaningful choices (Frankl, 1946/1986). By engaging in purposeful actions, forming meaningful connections, and choosing positive attitudes, people can find hope, self-esteem, and openness to new possibilities, all of which promote mental health and resilience (Erikson, 1964; Markstrom and Kalmanir, 2001; Search Institute, 2009; VanderVen, 2008). MMT emphasizes meaning in daily life through Frankl’s pathways of giving to the world (meaningful work, volunteering, giving to or helping others), experiencing in the world (secure attachment with other people, connection with something beyond oneself such as nature or the divine, engagement in personally meaningful leisure activities), and choosing attitudes (healthy thoughts and actions).

Differing from other evidence-based interventions, such as cognitive behavioral therapy, and mental health promotion programming that tends to target singular concerns, MMT is designed to address both internalizing and externalizing concerns, rather than a singular concern, as previously noted. This perspective aligns with cutting-edge protocols, such as the Unified Protocol, teaching skills that are relevant for a broad variety of concerns (Barlow et al., 2017). However, the Unified Protocol only addresses the broad spectrum of internalizing concerns, whereas MMT has been tested with both internalizing and externalizing symptoms. Specifically, in MMT, addressing unhelpful thoughts and behaviors is relevant for both internalizing and externalizing concerns, as is the ability to perspective-take, both for building attachment and developing empathy toward others. Experiencing a sense of meaning in daily life, and building that through gratitude exercises and purposeful engagement, is a buffer against mental illness symptoms (St John et al., 2024). Specifically, a sense of meaning mitigates against filling a meaning void with unhealthy behavioral substitutes for meaning, such as addictions or other unhelpful actions, and it reduces the risk of depression and anxiety (Flores, 2011; Frankl, 1946/1986). Further, as a PP3.0 theory, a sense of meaning in MMT allows for difficulties to be perceived as opportunities for growth (Lomas et al., 2020). Addressing the concept of meaning, MMT is the first to combine all of these theoretical foundations into a singular framework. A similar concept associated with hope, well-being, and resilience is hardiness (Bartone et al., 2022). By contrast to MM, which is considered to be teachable, hardiness is considered to be a fairly stable personality trait that is changeable only under some circumstances (Bartone et al., 2022). With hardiness, people have a sense of life commitment and feel a sense of meaning, experience a sense of control, present with openness to experience and perceive positives in the changes and challenges in life (Bartone et al., 2022). MMT asserts that these are teachable skills and are embedded in the DREAM program’s activities.

## DREAM program: developing resilience through emotions, attitudes and meaning

DREAM is a nationally-funded^1^, transdiagnostic mental health education program for children ages 7 to 10 that uses original songs, video webisodes, and hands-on activities (e.g., games, drama, art, read-aloud stories, discussions) to build skills for resilience. The hands-on activities themselves have enhanced meaning mindset and mental health in comparison to a control group, when administered by a clinician, which was comparable to the program with the video webisodes plus the hands-on activities that could be administered by teachers without a detailed manual (Potter, 2022).

DREAM modules are grounded in the PP3.0 MMT approach. The DREAM Program teaches children how to recognize emotions in themselves, in others, and in their bodies. It also teaches perspective-taking skills, healthy thinking, as well as calm-down and mindful behavioral strategies, and it emphasizes meaningful community and extracurricular engagement through the CHANGE model of MMT. The program consists of eight brief program modules, each including reinforcement activities. Stories, worksheets, examples, and discussion scenarios used throughout the program come directly from the research literature and lived experience of children. When administered in a classroom or community group, rather than by a therapist, the DREAM Program is educational, so children apply knowledge they have learned to the characters in songs, scenarios, or stories, rather than to their own issues. The overall goal is to build a “toolbox” of skills that children are able to use to promote their own meaning mindset and mental health.

### KTI approach to development

Over the past decade, a KTI approach (Armstrong et al., 2018) was used for ongoing co-creation in program development, refinement, and evaluation, as well as to mobilize knowledge directly to knowledge users. KTI is the research method of MMT, a participatory methodological approach (Armstrong et al., 2018). As previously noted, the overarching goal of a KTI approach is to promote the scientific utility standards, including credibility, acceptability, sustainability, and feasibility (Judd et al., 2001), as well as to adhere to a PP3.0 framework that incorporates diverse voices to ensure program fit (Lomas et al., 2020). KTI uses the main recommendations from Canadian Tri-council funding agencies to engage key knowledge users at all stages of program design and evaluation in order to enhance program fit (Tetroe et al., 2011). Specifically, to be credible, a program must do what it is designed to do quantitatively and qualitatively exhibit face-validity to the knowledge users (Trochim et al., 2015). To be acceptable, a program must integrate the scientific literature and be perceived by knowledge users to meet their needs (Judd et al., 2001). To be feasible, a program must be viewed as useable from a time and resource perspective (Judd et al., 2001). For a program to be sustainable, a program must be maintainable longer-term without requiring much external support (e.g., not requiring extensive or any “train-the-trainer” models; available to use long after the research funding ends; Lean and Colucci, 2013).

As KTI is a PP3.0 research methodology, its mixed methods framework acknowledges the groups and systems in which the research or program is embedded, it aims to be interdisciplinary, and it incorporates diverse voices to ensure the acceptability of the resultant programming for those whom it targets (Armstrong and Potter, 2023). For programs and research involving children, if young people are not involved in designing programs and research affecting them, then resulting services can fail to meet their needs (Amsden and VanWynsberghe, 2005; Commissioner for Children and Young People, 2016). Such services do not give rise to longer-term “action” because they are not acceptable to the group of young people whom they target (Amsden and VanWynsberghe, 2005). Therefore, DREAM program development and research has always built-in knowledge translation through co-creation with children and other knowledge users, such as mental health practitioners, teachers, and school board mental health teams.

### Findings

The DREAM Program has been evaluated over the past decade with school and community children, in comparison to control groups (Armstrong, 2017; Desson, 2018; Armstrong et al., 2018; Watt, 2020; Potter, 2022; Champaigne-Klassen, 2024). Qualitative findings regarding credibility, acceptability, feasibility, and sustainability were collected on an ongoing basis, and used for program development and refinement to meet the needs of children, as well as their parents, and teachers. When the program was first evaluated quantitatively with classroom groups of school children, it was found to significantly reduce stigma toward mental illness and help-seeking, with results yielding a medium effect size (Armstrong, 2017). Meaning mindset increased significantly from pre-test to post-test (agency, self-esteem, openness, hope; Armstrong, 2017). For the mental health education as usual control group from pre- to post-test, change was non-significant. In further research with intellectually gifted children at potential risk for mental health concerns due to emotional oversensitivities and other challenges, the program was found to enhance both meaning mindset and mental health from pre- to post-test (Armstrong et al., 2018). Further, the change in mental health scores was significantly predicted by a change in meaning mindset scores. Meaning mindset, as well as internalizing and externalizing mental health, were measured with the Child Identity and Purpose Questionnaire and the Interactive Symptom Assessment, respectively (Armstrong et al., 2019; Armstrong and Potter, 2022). For families on mental health waitlists (Watt, 2020), the program significantly enhanced child and parent internalizing and externalizing mental health, meaning mindset, and positive family functioning. A change in meaning mindset scores predicted mental health scores (Watt, 2020). Through virtual administration with school-aged children (Potter, 2022), both the English and French administration of the program enhanced meaning mindset, mental health, and positive COVID-19 coping. There was no significant difference between the program that could be teacher-lead with video webisodes to accompany the hands-on activities and the clinician-administered program (no webisodes, live presentation of mental health knowledge to accompany the hands-on activities). Thus, the program can be more widely distributed and administered by any community leader with the online webisodes and no training necessary. Online, with families of intellectually gifted children (Champaigne-Klassen, 2024), the program enhanced meaning mindset and mental health for both children and parents. Further, meaning mindset negated—fully mediated—the relationship between the challenges associated with giftedness (oversensitivities, perfectionism, asynchronous development) and mental health concerns.

### DREAM modules

For the modules, there are suggested and tested adaptations for neurodiverse children or those with behavior challenges so that activities can be safe, engaging, and helpful for all participants. The activities listed below are the standard administration. Each of the modules below also include music videos and discussions to reinforce learning taught in the educational webisodes and hands-on activities.

#### Module 1

In the first module, children learn about what mental health and mental illness are in order to reduce stigma. They are taught that there are things they can do to help themselves feel better and how to get help. They also learn about different, common feelings and what those might look like. Experientially, to learn about different, common feelings and how to recognize these feelings in themselves and others, children or family groups play a feelings improvisation game, acting out different emotions that are guessed by their classmates, the group of participants in the community, or by family members, depending on who is participating in the program. At a later time, there is a reinforcement matching game activity where they match the feeling faces to the feeling words.

#### Module 2

In the second module, additional common feelings are taught. Children also learn about how feelings are affected by thinking and behavior and how thinking and behavior affect feelings. They are taught about different ways people can feel in response to the same situation. Experientially, children learn about the connection between thoughts and feelings through an “emotions go fish” game. When children get a matching pair of feelings cards, they describe a situation in which children might feel that feeling. The classroom or the family then brainstorms different ways people might feel in the same situation and why people might feel differently. The later reinforcement activity involves a scenario worksheet where the group explores different ways people might feel in the presented situations and why.

#### Module 3

Children are taught mindful relaxation techniques, how to create a worry time, and why good sleep hygiene is important. Experientially, children engage in a feelings drama where they walk across a room acting out a little bit of a feeling (anger), more of the feeling, a whole lot of the feeling, noting each time where in their body they feel that feeling. They then walk it back to just acting out a little bit of that feeling. They learn to recognize when they have just a little bit of a feeling in their body so they can use calm down strategies more effectively. Children practice a grounding exercise—5 things they see, 4 things they can touch, 3 things they hear, 2 things they smell, 1 big deep breath—and a mindful five-finger breathing exercise. Later in the week, they do an imagery drawing relaxation activity (i.e., draw a relaxing scene and imagine all the things in that scene that they would see, hear, touch, feel (emotionally), smell, and potentially taste).

#### Module 4

Children learn about feelings as helpful signals: Joy is a helpful signal that the activity one is doing is meaningful, fear could be an alarm bell for real danger, or sometimes one may simply be having negative thoughts and the feelings could indicate this. Children learn about what happens with avoidance versus acting in a helpful way in response to feeling signals. Children participate in an experiential activity in which they are presented with a frustrating story, then they play a game with balloon or balls (or a paper ball, if virtual) to show them how brief distraction can help mood or be calming. The class or family then generate a list of brief feel-good/calm-down activities. Later in the week, as a reinforcement activity, children review the list of feel good/calm down activities and pick ones that they think could be helpful for them to try.

#### Module 5

The connection between thoughts and feelings happens in depth in this module. Children learn how to be a thought detective and question their thoughts. They also learn about what gratitude means and its connection to well-being. There is an experiential activity where children make a “crown of thoughts” and they a given a scenario with an unhelpful thought to stick to their crown. They show others what their feeling face would look like if they had this thought. In groups, children question the thought and come up with a new, more helpful thought to stick to their crown of thoughts and make the new feeling face. The reinforcement activity is a “stinky thought” worksheet where children think of how someone could feel if they had the unhelpful thought. Then, they discuss different ways that someone could feel in the same situation and how feelings could differ as a result.

#### Module 6

Children learn how to reframe thoughts about situations that concern them. They learn about the problem with avoidance. They are taught a three-step model to identify feelings, question their thoughts, and how to choose a helpful action. Through the educational webisode video, storytelling, and role-playing scenarios, children also learn informal conflict resolution skills, when to talk to a peer about their concerns, or when to talk to a trusted adult. They learn to apply the “EASE” conflict coaching model—Empathize, Acknowledge, Solve, and Evaluate (Armstrong and Epperson, 2024; Epperson and Armstrong, 2024) to maintain healthy interactions with their peers:

- Empathize (Empathic listening): Empathy → Child “A” states the problem and their feeling using “I” statements (non-blaming language). For example, Child A says: “I was feeling left out at recess when everyone disappeared and went outside when I was getting my coat on.” Then Child B repeats back what they heard: “Everyone disappeared and went outside….” Child B considers and labels the feeling the other person is having: “And you were feeling left out.” Child A acknowledges that this is what they said/felt, or clarifies: “Yes, everyone went outside without me. I could not find you for a while and felt left out.”
- Acknowledge (Acknowledge circumstances): Set goal → The children explore things that can change, rather than the things that cannot be changed. They focus on small, realistic goals that they both agree on and identify ways they would like things to be different next time. For example, Child A says: “I’d like to come up with a way that no one gets left behind.” Child B replies: “That’s a good goal.”
- Solve (Sensible action): Action plan → The children explore actions that could be taken to meet the goal. Child A might say: “I’d like for everyone to check if we are all going outside together so that no one is left behind.” Child B reflects what they hear, and both children agree on an action. For example, Child B says: “We could all look to see that everyone is ready and wait at the door as a group to go out when everyone is there.”
- Evaluate (Explore feelings and thoughts): Prepare for action → The children look at how everyone feels about the plan. Child A: “That sounds like a good plan, let us do it! I feel happy about it.” Child B: “Me too!”

Learning healthy, informal conflict resolution skills in childhood is crucial for social and emotional development. These skills help children navigate disagreements constructively, fostering empathy and effective communication skills. Early mastery of conflict resolution promotes a positive self-image and reduces the likelihood of aggressive behaviors. Learning to apply the EASE model in childhood conflicts lays the foundation for healthy relationships and collaborative problem-solving in adulthood.

#### Module 7

Children learn about purpose-driven activities, cultivating a meaning mindset through the following avenues from our Ontario Ministry of Education Resilience Brief (Armstrong et al., 2018):

- Belonging: connecting with others, feeling valued by others, forming relationships, contributing to the community or to a group, connecting to the natural world
- Well-being: maintaining physical and mental health through self-care, sense of self, and self-regulation skills
- Engagement: being involved and focused, curious, and open, thereby developing problem-solving and creative-thinking skills
- Expression or communication: communicating and listening to others, developing the ability to convey information, ideas, and feelings through actions or words

Children also learn about the relationship between extracurricular or leisure activities and well-being. They are taught about the difference between passive (e.g., some types of screen time) and active leisure. Experientially, they participate in a card-making activity, making a card for someone they appreciate, but they do not have to give this card to them. They explore how the other person would feel if this card were received and how they would then feel themselves. They brainstorm things they could do for others at home, at school, or in the world to help others feel good and, therefore, feel good themselves. As a reinforcement activity, they participate in a “fun activities card sort” game exploring common at-school or at-home activities they might enjoy participating in regularly to have things to look forward to.

#### Module 8

Children learn about the problems with emotional avoidance versus listening to what their feeling signals are trying to tell them. They also learn about grit: when to move on from a situation versus when to see challenges as an opportunity for growth and what can be learned from difficult situations (e.g., losing a hockey game, an assignment does not go as well as they would like: What did they like about what they did? What could they do different next time?”). Building on all the skills they learned across the modules, they learn how to cope with major life difficulties. All the major concepts in the program are summarized through superhero comics. Being their own superheroes, the children learn how to apply all the skills they have learned to solving the problems in comic drawings and finishing the drawings with stick figures, healthy thoughts, healthy, actions, and feeling faces in response.

For all of the activities in this mental health education protocol, exercises are applied to characters in stories, songs, and scenarios, rather than to personal issues. This ethical consideration allows for program delivery that mitigates the potential risk of teachers or parents having to address children’s discussion of their own issues. Collaborative development of DREAM with four school boards has allowed for the development of a protocol that school board mental health teams and university and school board ethics committees believe minimizes potential harm of mental health teaching and maximizes benefit.

Overall, the DREAM Program’s transdiagnostic approach to mental health promotion in children teaches strategies to regulate emotions through recognizing and responding to feelings, tolerating distress, and enhancing social skills through perspective-taking, managing conflict, help-seeking, and using empathy. The program enhances agency over thoughts and behaviors, openness to learning, to new experiences, and to feelings, positive self-concept to set and reach goals, as well as positive *social* self-concept, and hope for the future. As noted previously, the inverse of these sources of resilience underlies many mental health concerns. Further research should consider the long-term impact of the DREAM program. Moreover, although the DREAM program has been administered to diverse samples, it will be important to explore specific diverse populations (e.g., new immigrants, children on the Autism spectrum, gender diverse children and youth) to enhance its applicability to diverse populations. Currently, graduate thesis students are researching MMT through the EASE conflict coaching protocol in building a therapeutic or ombuds alliance with visible minority adults in psychotherapy and those who have accessed ombuds services.

## Conclusion

Recently, there has been an emerging consensus that the prevention and treatment of singular diagnostic concerns may not be as beneficial as taking an approach that cuts across diagnostic boundaries, or targeting factors that underlie many internalizing and externalizing symptoms (Dalgleish et al., 2020). In fact, it seems that transdiagnostic approaches to conceptualization of distress may better represent the clinical reality and complexity of mental health concerns (Dalgleish et al., 2020). Beyond the therapeutic alliance in successful treatment outcomes, there are common factors in different evidence-based treatments that can lead to positive well-being across different diagnostic profiles (Cuijpers et al., 2019; Dalgleish et al., 2020).

Using a MM theoretical framework (or Meaning Mindset Therapy), the DREAM Program protocol serves as a testable, transdiagnostic model for both mental health education, where it has been primarily used, and for child or family therapy. To date, the model has been tested with waitlist controls and with “mental health education as usual” controls. Further, the measures used to assess the program are in themselves transdiagnostic, including meaning mindset—measuring agency, openness, self-concept and hope—and general internalizing and externalizing mental health. Consistent with recommendations for transdiagnostic mental health (Dalgleish et al., 2020), these measures assess each aspect of well-being on a continuum from healthy to problematic. Thus, in practice monitoring, these measures (the Child Identity and Purpose Questionnaire and the Interactive Symptom Assessment) may be helpful complements to a transdiagnostic approach. Today, MMT is one of the first transdiagnostic approaches for children and also one of the first to be relevant for both internalizing and externalizing mental health issues. Future research should involve testing MMT using the DREAM protocol in therapy settings compared to standard manualized treatments for singular diagnostic concerns. With further research, MMT may serve to advance transdiagnostic approaches for mental health education and treatment for Canadian children and families and beyond, addressing core challenges associated with both internalizing and externalizing concerns.

## Footnotes

^1^Funded by the Canadian Social Sciences and Humanities Research Council Insight Development & Insight Grants.
